# Sirtuin 5-mediated desuccinylation of Slc25a4 inhibits osteoporosis by enhancing mitochondrial respiration

**DOI:** 10.1038/s41413-025-00464-7

**Published:** 2025-11-17

**Authors:** Jun Chen, Xinquan Jiang

**Affiliations:** 1 Department of Prosthodontics, Shanghai Ninth People’s Hospital, Shanghai Jiao Tong University School of Medicine, Shanghai, China; 2 Shanghai Stomatological Hospital, Fudan University, Shanghai, China; 3 College of Stomatology, Shanghai Jiao Tong University, Shanghai, China; 4 National Center for Stomatology, National Clinical Research Center for Oral Diseases, Shanghai Key Laboratory of Stomatology, Shanghai Research Institute of Stomatology, Shanghai Engineering Research Center of Advanced Dental Technology and Materials, Shanghai, China

**Keywords:** Calcium and phosphate metabolic disorders, Bone quality and biomechanics

## Abstract

Osteoporosis is a prevalent metabolic bone disorder that develops when osteoclast-mediated bone resorption chronically exceeds osteoblast-driven bone formation. The molecular pathways that govern osteogenic dysfunction and connect cellular metabolism to differentiation regulation remain poorly characterized. Here, we identify Sirtuin 5 (Sirt5) as a pivotal osteogenic regulator through bioinformatic screening and functional validation in *Sirt5*-knockout mice. Mechanistically, Sirt5 governs mitochondrial homeostasis by desuccinylating Solute Carrier Family 25 Member 4 (Slc25a4) at lysine 147 (K147), as demonstrated by quantitative succinylome profiling and site-directed mutagenesis. This site-specific desuccinylation triggers Slc25a4 degradation, attenuating mitochondrial oxidative stress and promoting osteoblast differentiation. Crucially, Slc25a4-K147 succinylation drives osteoporosis progression, while Sirt5-mediated desuccinylation at this site confers protection. Our work reveals the Sirt5-Slc25a4-K147 axis as a novel regulatory mechanism coupling mitochondrial metabolism to bone homeostasis, offering a therapeutic target for osteoporosis intervention.

## Introduction

Osteoporosis is a prevalent metabolic bone disease characterized by decreased bone mass and microstructural deterioration, resulting in bone fragility and elevated fracture risk.^1^ This disease substantially compromises patients’ quality of life and imposes a significant economic burden.^2^ The pathogenesis of osteoporosis centers on an imbalance in bone remodeling, with bone resorption exceeding bone formation.^3^ Enhancing osteoblast activity and restoring bone remodeling balance are critical for osteoporosis prevention and treatment.^4^ Osteoblast activity is modulated by multiple signaling pathways, including Wnt/β-catenin, BMP/Smad, and Notch.^5–8^ Although significant progress has elucidated genes regulating osteogenic differentiation, the complexity of these regulatory networks makes identifying key osteogenic regulators in osteoporosis challenging. Thus, discovering these essential genes is crucial to deciphering osteoporosis pathogenesis and advancing therapeutic development.

Osteogenesis is intimately associated with mitochondrial function.^9^ Mitochondria play pivotal roles in energy production via oxidative phosphorylation and in mediating cellular signaling.^10^ In osteoblasts, optimal mitochondrial function is required to generate ATP for energy-intensive processes including collagen synthesis and matrix mineralization.^11,12^ Moreover, mitochondria maintain reactive oxygen species (ROS) levels homeostasis - physiological ROS levels promote osteoblast differentiation, whereas excessive ROS induces oxidative stress and impairs cellular function.^13,14^ Sirt5, an NAD^+^-dependent deacylase, modulates mitochondrial function through deacetylating and demalonylating mitochondrial proteins, optimizing their activity and stability.^15–17^ Emerging evidence suggests Sirt5 modulates osteoblast function and bone formation, highlighting its potential as a novel therapeutic target in osteoporosis management.

In this study, we identified Sirt5 as a critical regulator of osteogenic differentiation in osteoporosis through comprehensive data mining of osteogenesis- and osteoporosis-related expression profiles. Genetic ablation of Sirt5 confirmed its protective role against osteoporosis. Vitro experiments demonstrated that Sirt5 promotes osteogenesis via modulation of mitochondrial respiration. Mechanistically, we identified and functionally validated Sirt5’s target proteins through desuccinylation profiling. These findings establish new theoretical targets and provide experimental evidence for osteoporosis intervention. Notably, our results demonstrate the pivotal role of Sirt5-mediated mitochondrial regulation, underscoring its therapeutic potential for osteoporosis treatment.

## Results

### SIRT5 was identified as a key regulator of osteogenesis in osteoporosis

**Fig. 1:**
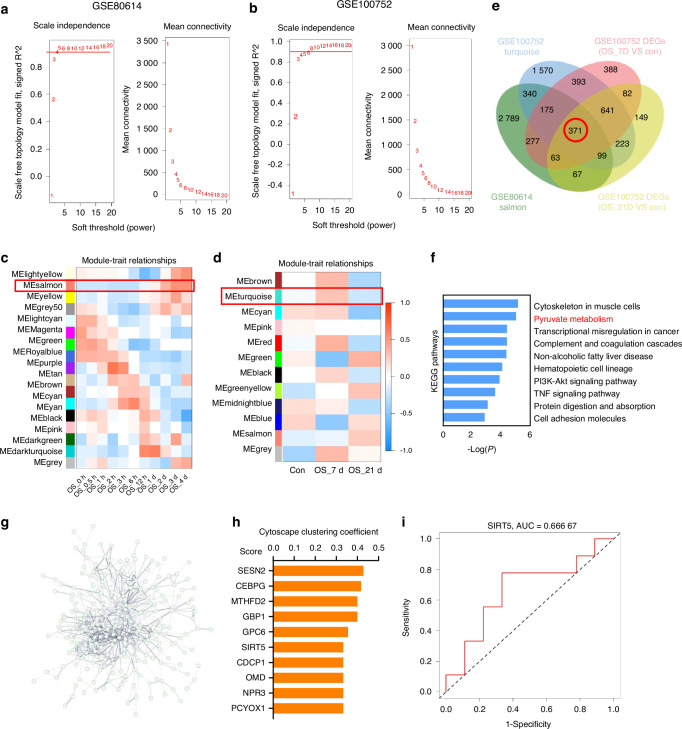
SIRT5 was identified as a key gene regulating osteogenesis. **a**, **b** Scale-free fit index analysis for determining soft-thresholding power in the GSE80614 and GSE100752 datasets. **c**, **d** Module-trait relationships in co-expression networks. **e** Venn diagram showing overlapping genes between the turquoise module from the GSE100752 dataset, the salmon module from the GSE80614 dataset, and DEGs from the GSE100752 dataset (OS_7D vs. Con and OS_21D vs. Con). **f** KEGG pathway enrichment of intersecting genes, highlighting key osteogenic differentiation pathways. **g** Protein-protein interaction network of overlapping genes, constructed using STRING. **h** Top 10 genes ranked by clustering coefficient in the interaction network. **i** ROC curve evaluating the predictive performance of SIRT5 expression for disease status from the GSE30159 dataset

To identify key osteogenic regulators in osteoporosis, we conducted Weighted Gene Co-expression Network Analysis (WGCNA) on the GSE80614^18^ and GSE100752^19^ datasets, which profiled gene expression in Bone Marrow Mesenchymal Stem Cells (BMSCs) during osteogenic differentiation. After quality control and outlier removal (Fig. S1a, b), we established co-expression networks using a soft-power threshold of 4 (scale independence: 0.85 for GSE80614; 0.9 for GSE100752; Fig. 1a, b). This revealed 17 modules in GSE80614 and 12 in GSE100752 (Fig. 1c, d), with Topological Overlap Matrix (TOM) heatmaps demonstrating module relationships through connectivity gradients (Fig. S1e, f). The salmon module in GSE80614 and the turquoise module in GSE100752 showed the strongest correlation with osteogenesis (Fig. S1g, h). Their expression levels are depicted in Fig. S1i, j.

We identified stage-specific differentially expressed genes (DEGs) in the GSE100752 dataset by comparing osteogenic differentiation at early (OS_7D vs control) and late (OS_21D vs control) stages (Fig. S2a, d). Integrative analysis of the overlapping genes from the GSE80614 salmon module, GSE100752 turquoise module, and stage-specific DEGs (Fig. 1e), especially those shown in the Venn diagram, revealed pyruvate metabolism as the most significantly enriched pathway through KEGG analysis (Fig. 1f). Protein-protein interaction network construction using STRING database (https://cn.string-db.org/) (Fig. 1g) and clustering coefficient analysis identified top hub genes (Fig. 1h), among which ROC curve analysis of the GSE30159^20^ dataset demonstrated SIRT5 and NPR3 as significant osteoporosis risk predictors (Fig. 1i, Fig. S3a). Given SIRT5’s mitochondrial regulatory role, we confirmed its significant downregulation in ovariectomized (OVX) mouse bone tissue versus Sham controls (Fig. S2e), establishing SIRT5 as a master regulator of osteoporosis pathogenesis through pyruvate metabolism modulation.

### Sirt5 deficiency exacerbates osteoporosis by impairing bone formation

**Fig. 2:**
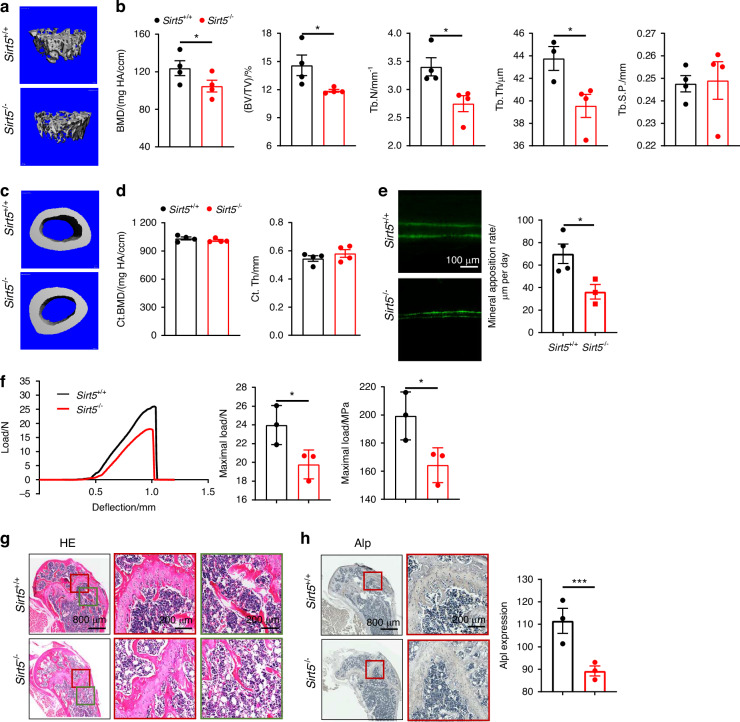
Sirt5 knockout induced osteoporosis in mice. **a** 3D micro-CT reconstruction images of trabecular bone in *Sirt5*^+/+^ and *Sirt5*^-/-^ mice. **b** Quantitative microarchitectural parameters of micro-CT: BMD, BV/TV, Tb.N., Tb.Th., and Tb.Sp. (n = 4) **c** 3D micro-CT reconstruction images of cortical bone. **d** Quantitative microarchitectural parameters of micro-CT: Ct.Th. and Ct.BMD. **e** Osteogenic activity in the femora, represented by calcein staining. **f** Left: Representative load-deflection diagram from a three-point bending test performed on femora. Middle and Right: Maximum load measured during the test. **g**, **h** Femoral analysis showing H&E staining and Alp immunohistochemistry at both low (800 μm) and high (200 μm) magnification scales. Data represent mean ± SEM in (**b**, **d**, **f**, **h**). Statistics used an unpaired two-tailed *t*-test (**b**, **d**, **f**, **h**). Significance is noted as *P < 0.05, ***P < 0.001

To investigate Sirt5’s role in bone homeostasis, we established an OVX-induced osteoporosis model using 4-week-old female *Sirt5* knockout mice^21^ (*Sirt5*^-/-^) and wild-type (*Sirt5*^+/+^) mice. After 30 days, femora were harvested for micro-computed tomography (μCT) analysis. *Sirt5*^-/-^ mice showed significant reductions in trabecular mass and bone formation rate compared to *Sirt5*^+/+^ mice (Fig. 2a). These findings were supported by quantitative μCT parameters: bone volume/total volume (BV/TV), trabecular thickness (Tb.Th.), number (Tb.N.), and separation (Tb.Sp.) (Fig. 2b). But cortical bone mass remained unchanged (Fig. 2c, d). Calcein labeling revealed impaired cortical bone formation in *Sirt5*^-/-^ mice (Fig. 2e). Three-point bending tests further demonstrated reduced bone stiffness in the femora of *Sirt5*^-/-^ mice compared to their *Sirt5*^+/+^ counterparts (Fig. 2f). Hematoxylin and eosin (H&E) staining confirmed trabecular bone loss in *Sirt5*^-/-^ mice (Fig. 2g). Alp expression was significantly reduced in *Sirt5*^-/-^ mice by immunohistochemistry (Fig. 2h). These results demonstrate that Sirt5 deficiency exacerbates osteoporosis by impairing bone formation, highlighting its regulatory role in bone homeostasis.

### Sirt5 promotes osteogenic differentiation through mitochondrial respiration regulation

**Fig. 3:**
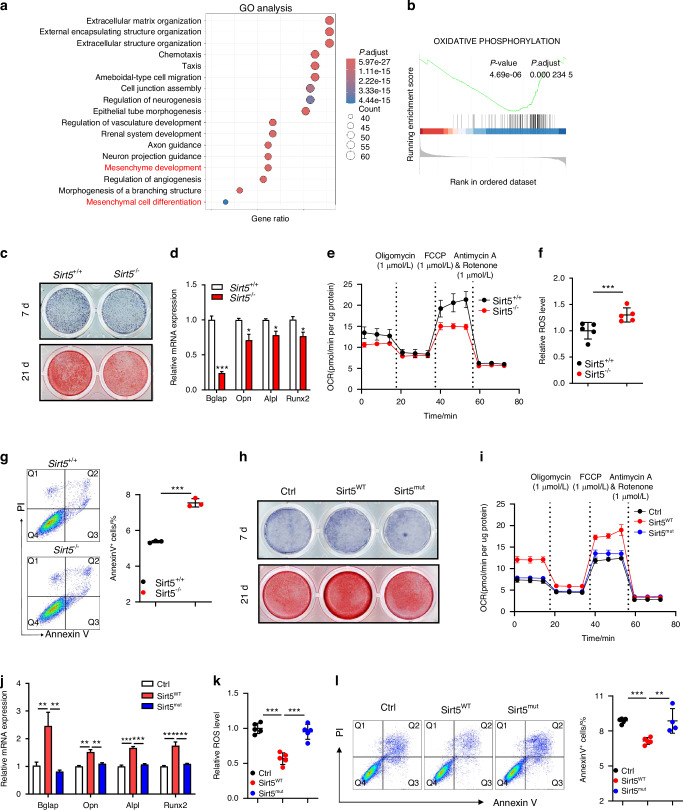
Sirt5 enhances osteogenesis and inhibits apoptosis by facilitating mitochondrial respiration. **a** GO analysis of hallmark gene sets in BMSCs osteogenically differentiated for 7 days in *Sirt5*^+/+^ and *Sirt5*^-/-^ mice. (n = 3) **b** GSEA analysis of DEGs. **c** Upper: Alp staining in BMSCs after 7-day osteogenic differentiation. Lower: Alizarin red staining of BMSCs after 21-day differentiation. In *Sirt5*^+/+^ and *Sirt5*^-/-^ BMSCs after 7-day osteogenic differentiation: Relative mRNA levels of osteogenic markers (*Bglap, Opn, Alpl, Runx2*; **d**), mitochondrial respiration (OCR; **e**), intracellular ROS levels (**f**), and apoptosis analysis by flow cytometry (**g**). **h** Upper: Alp staining in BMSCs overexpressing Ctrl, Sirt5^WT^, or Sirt5^mut^ after 7-day osteogenic differentiation. Lower: Alizarin red staining after 21-day differentiation. In BMSCs overexpressing Ctrl, Sirt5^WT^, or Sirt5^mut^ after 7-day osteogenic differentiation: Relative mRNA levels of osteogenic markers (**i**), mitochondrial respiration (OCR; **j**), intracellular ROS levels (**k**), and apoptosis analysis by flow cytometry (**l**). Data represent mean ± SEM in (**d**–**g**) and (**i**–**l**). Statistics used an unpaired two-tailed *t*-test (**d**–**g** and **i**–**l**). Significance is noted as *P < 0.05, **P < 0.01, ***P < 0.001

To investigate Sirt5’s role in osteogenesis, we compared transcriptomes of *Sirt5*^+/+^ and *Sirt5*^-/-^ BMSCs following 7-day osteogenic induction. Unsupervised clustering of DEGs revealed distinct transcriptional profiles (Fig. S4a). Gene Ontology (GO) analysis identified significant enrichment of terms related to mesenchymal differentiation (Fig. 3a). Gene Set Enrichment Analysis (GSEA) demonstrated significant suppression of oxidative phosphorylation pathways in *Sirt5*^-/-^ compared to *Sirt5*^+/+^ BMSCs (Fig. 3b and Fig. S4b), linking Sirt5-dependent mitochondrial respiration to osteogenic regulation. After 7-day differentiation, Alp staining and activity assays showed a reduced number of Alp^+^ osteoblasts in the *Sirt5*^-/-^ group (Fig. 3c and Fig. S4c). Similarly, 21-day alizarin red staining showed diminished calcium nodule formation in *Sirt5*^-/-^ cells (Fig. 3c and Fig. S4c). Expression levels of osteogenic markers (*Bglap, Opn, Alpl*, and *Runx2*) were significantly downregulated in the *Sirt5*^-/-^ group (Fig. 3d). Our results indicate that Sirt5 promotes BMSCs osteogenesis, which is associated with mitochondrial respiration.

Given Sirt5’s role in mitochondrial oxidative phosphorylation, we performed Seahorse XF metabolic assays. *Sirt5*^-/-^ BMSCs showed significantly impaired mitochondrial function, including ATP production, basal respiration, and maximum respiration compared to the *Sirt5*^+/+^ BMSCs (Fig. 3e and Fig. S4d). As mitochondrial dysfunction frequently induces ROS accumulation,^22^ we next measured intracellular ROS levels, observing an increase in the *Sirt5*^-/-^ BMSCs (Fig. 3f). Consistent with prior reports that ROS suppresses osteogenic differentiation,^23,24^ our findings suggest Sirt5 deficiency impairs osteogenesis through mitochondrial dysfunction-induced ROS overproduction. Mechanistically, ROS can open mitochondrial permeability transition pores, activate caspases, dysregulate calcium homeostasis, and trigger apoptosis.^25^ To assess the percentage of apoptotic cells, we performed flow cytometry analysis, which revealed a significant increase in the proportion of apoptotic cells in the *Sirt5*^-/-^ group (Fig. 3g). Collectively, these data demonstrate that Sirt5 deficiency disrupts mitochondrial respiration, leading to ROS accumulation that both impairs osteogenesis and promotes apoptosis.

As an NAD^+^-dependent deacylase, SIRT5 mediates lysine deglutamylation, desuccinylation, and depropionylation to regulate cellular processes.^26^ To determine the functional dependence of Sirt5-mediated osteogenesis on its enzymatic activity, we generated a catalytically inactive mutant (H158Y).^27^ BMSCs were isolated and transduced to express either control (Ctrl), Sirt5 wild-type (Sirt5^WT^), or Sirt5 mutant (Sirt5^mut^) (Fig. S4e). After 7-day osteogenic induction, Sirt5^WT^ significantly increased Alp^+^ osteoblast numbers and activity while Sirt5^mut^ showed no effect, with similar results observed for mineralization at day 21 (Fig. 3h and Fig. S4f). Consistently, Sirt5^WT^ specifically upregulated osteogenic markers at mRNA level (Fig. 3i). These data conclusively demonstrate that Sirt5’s pro-osteogenic effects strictly depend on its catalytic activity. Seahorse XF assay showed Sirt5^WT^-specific enhancements in mitochondrial respiration (Fig. 3j and Fig. S4g), reduced intracellular ROS versus control (Fig. 3k). Flow cytometry confirmed a reduction in apoptosis with Sirt5^WT^ overexpression, while Sirt5^mut^ showed no protective effect (Fig. 3i). These results demonstrate that Sirt5 promotes osteogenesis through its catalytic activity-dependent regulation of mitochondrial function, which maintains redox homeostasis and supports cell survival during bone formation.

### Sirt5-mediated desuccinylation of Slc25a4 at lysine 147 induces its degradation

**Fig. 4:**
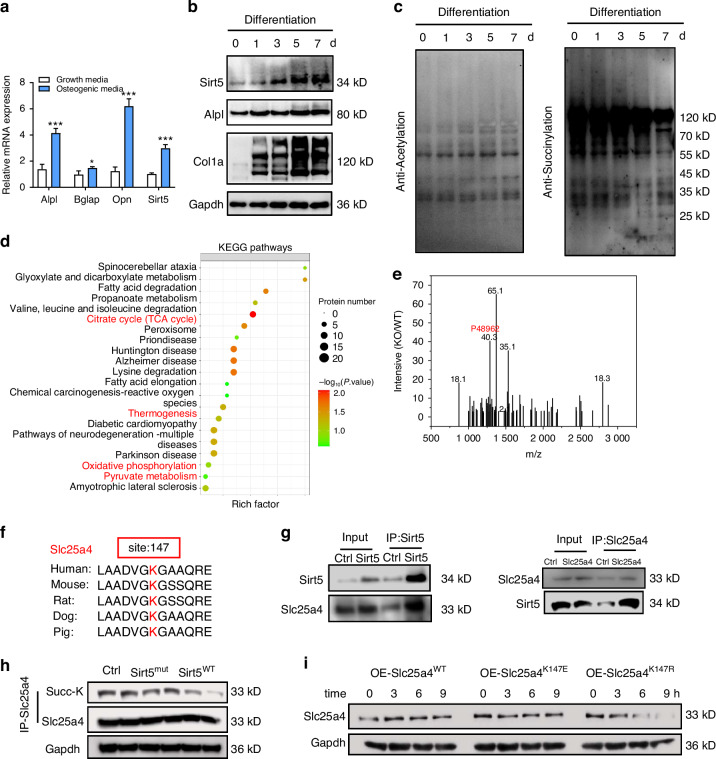
Sirt5 desuccinylates the K147 site of Slc25a4 leading to degradation. **a** Relative expression levels of *Alpl, Bglap, Opn*, and *Sirt5* in BMSCs treated with growth media or osteogenic media for 7 days. **b**, **c** Immunoblot analysis of osteogenic markers (Alpl, Col1a1, with Gapdh; **b**) and protein post-translational modifications (acetylation, and succinylation; **c**). **d** KEGG analysis of proteins from the Venn diagram intersection. **e** The LC-MS/MS spectrum of succinylated proteins. **f** Evolutionary conservation analysis of the Slc25a4 K147 site across species. **g** Left: Immunoblotting against Sirt5 and Slc25a4 in whole cell lysates and Sirt5-immunoprecipitated eluates. Right: Immunoblotting against Slc25a4 and Sirt5 in whole cell lysates and Slc25a4-immunoprecipitated eluates. **h** Immunoblotting against Succ-K, Slc25a4, and Gapdh in whole cell lysates and Slc25a4-immunoprecipitated eluates. **i** Immunoblotting against Slc25a4 and Gapdh in whole cell lysates expressing Slc25a4^WT^, Slc25a4^K147E^, or Slc25a4^K147R^ after treatment with CHX for 0, 3, 6, and 9 h. Data represent mean ± SEM in a. Statistics used an unpaired two-tailed *t*-test (**a**). Significance is noted as *P < 0.05, ***P < 0.001

To elucidate the molecular mechanisms underlying Sirt5’s regulation of osteogenesis, we investigated its temporal expression pattern and substrate specificity during BMSCs differentiation. Time-course analysis revealed progressive upregulation of Sirt5 alongside osteogenic markers Col1a1 and Alpl (Fig. 4a, b and Fig. S5a). Pan-lysine modification analysis showed global protein succinylation decreased by day 7, but acetylation levels remained stable (Fig. 4c). Next, we performed liquid chromatography-tandem mass spectrometry (LC-MS/MS) to identify the target proteins of Sirt5. Quantitative analysis of succinylated proteins showed significant differences between *Sirt5*^-/-^ and *Sirt5*^+/+^ BMSCs (Fig. S5b–d). Heatmap illustrates the proteins with significant alterations in succinylation levels in *Sirt5*^-/-^ BMSCs (Fig. S5e). KEGG pathway enrichment of identified proteins indicated substantial alterations in several mitochondria-associated pathways, including the citrate cycle (TCA cycle), thermogenesis, oxidative phosphorylation, and pyruvate metabolism (Fig. 4d). GO analysis confirmed significant changes in mitochondrial function (Fig. S6a–c). Together, these findings demonstrate that Sirt5 orchestrates osteogenic differentiation through selective desuccinylation of mitochondrial proteins, thereby modulating cellular metabolism to promote bone formation.

To identify the key substrates of Sirt5-mediated desuccinylation, we established a protein-protein interaction network (Fig. S4d). Comparative analysis revealed markedly increased succinylation of Slc25a4 (ID: P48962) at lysine 147 (K147) in *Sirt5*^-/-^ BMSCs (Fig. 4e). As an integral component of the mitochondrial inner membrane, Slc25a4 facilitates the bidirectional transport of ADP and ATP across mitochondrial membranes, thereby coupling cytosolic and matrix nucleotide pools.^28,29^ This nucleotide exchange is essential for sustaining mitochondrial oxidative phosphorylation and cellular energy homeostasis. Additionally, Slc25a4 modulates apoptotic signaling through regulation of the mitochondrial permeability transition pore.^30^ Impaired Slc25a4 function promotes mitochondrial oxidative stress, ultimately resulting in genomic instability and cellular compromise.^31,32^ These findings position Slc25a4 as a pivotal effector linking Sirt5-mediated desuccinylation to the regulation of mitochondrial dynamics and osteogenic differentiation.

Our study characterized the functional regulation of Slc25a4 by succinylation. Evolutionary analysis revealed striking conservation of the K147 residue across species (Fig. 4f). Co-immunoprecipitation (co-IP) confirmed a direct physical interaction between Sirt5 and Slc25a4 (Fig. 4g). Following the overexpression of Ctrl, Sirt5^WT^, or Sirt5^mut^, we immunoprecipitated Slc25a4 and quantified its succinylation status. Immunoblot analysis showed that Sirt5^WT^, but not Sirt5^mut^, effectively decreased Slc25a4 succinylation (Fig. 4h and Fig. S6e). To mechanistically dissect this regulatory process, we generated site-directed mutants of Slc25a4 at K147, creating Slc25a4^K147R^ and Slc25a4^K147E^ variants. The Slc25a4^K147E^ mutation substitutes lysine with glutamic acid to mimic the succinylated state, while Slc25a4^K147R^ replaces lysine with arginine to simulate the desuccinylated conformation. Following transfection with these mutants, cells were treated with cycloheximide (CHX) for 0, 3, 6, or 9 h to monitor protein turnover. Protein stability assays revealed accelerated degradation of the Slc25a4^K147R^ mutant relative to the Slc25a4^K147E^ variant (Fig. 4i and Fig. S6f). These data suggest that Sirt5 promotes Slc25a4 degradation through its enzymatic activity in removing the succinylation modification at K147.

### Slc25a4 K147 succinylation impairs bone formation and mitochondrial function

**Fig. 5:**
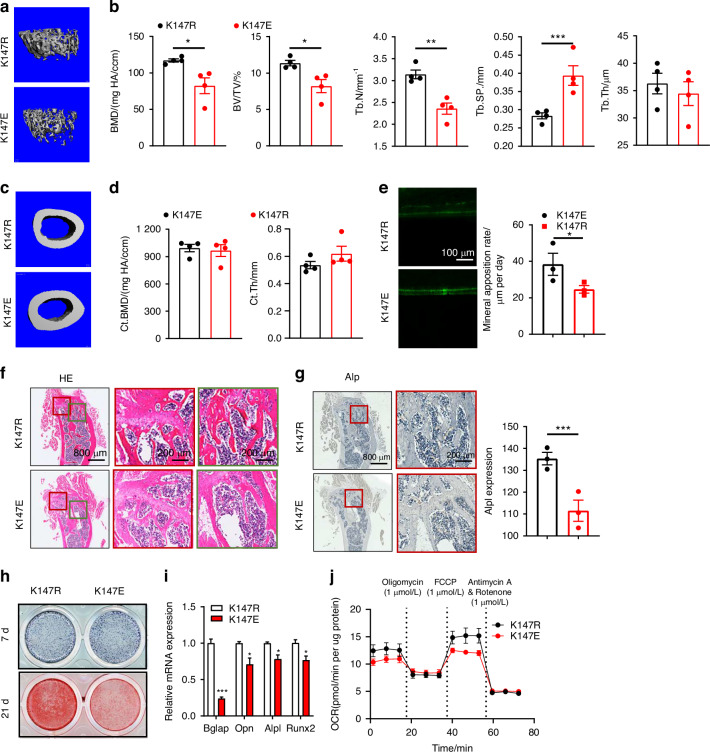
Slc25a4 K147 succinylation inhibits osteogenesis and mitochondrial respiration. **a** 3D micro-CT reconstruction images of trabecular bone in OE-Slc25a4^K147R^ or OE- Slc25a4^K147E^ mice. **b** Quantitative microarchitectural parameters of micro-CT. (n = 4) **c** 3D micro-CT reconstruction images of cortical bone. **d** Quantitative microarchitectural parameters of micro-CT. **e** Osteogenic activity in the femora, represented by calcein staining. **f**, **g** Femoral analysis showing H&E staining and Alp immunohistochemistry at both low (800 μm) and high (200 μm) magnification scales. **h** Upper: Alp staining in BMSCs overexpressing Slc25a4^K147R^ or Slc25a4^K147E^ after 7-day osteogenic differentiation. Lower: Alizarin red staining after 21-day osteogenic differentiation. **i**, **j** Relative mRNA levels of osteogenic markers (**i**), mitochondrial respiration (OCR; **j**). Data represent mean ± SEM in (**b**, **d**, **e**, **g**, **i**). Statistics used an unpaired two-tailed *t*-test (**b**, **d**, **e**, **g**, **i**). Significance is noted as *P < 0.05, **P < 0.01, ***P < 0.001

To examine the role of Slc25a4 K147 succinylation in osteogenesis, we employed AAV vectors to deliver Slc25a4^K147E^ or Slc25a4^K147R^ variants via intrathecal injection, enabling retrograde transport to bone marrow. 7 days post-injection, femora analysis confirmed overexpression of both variants, with Slc25a4^K147E^ showing specific elevation in Slc25a4 succinylation (Fig. S7a). After a 30-day feeding period, the femora were collected for micro-CT analysis. Quantitative μCT demonstrated significantly lower trabecular bone volume fraction (BV/TV) and bone formation rate in Slc25a4^K147E^ mice versus Slc25a4^K147R^ controls (Fig. 5a, b). Cortical bone parameters remained unchanged between groups (Fig. 5c, d). Dynamic histomorphometry via calcein double-labeling showed impaired cortical mineralization in Slc25a4^K147E^ mice (Fig. 5e). Histological analysis confirmed substantial reductions in trabecular number and thickness in Slc25a4^K147E^ mice (Fig. 5f). Immunohistochemistry revealed diminished Alp expression in Slc25a4^K147E^ bone sections (Fig. 5g). These findings demonstrate that site-specific succinylation of Slc25a4 at K147 directly compromises osteogenic potential and drives osteoporosis pathogenesis through impaired bone formation.

To determine the functional impact of Slc25a4 K147 succinylation on osteogenesis, we overexpressed Slc25a4^K147R^ or Slc25a4^K147E^ in BMSCs. Following 7-day osteogenic induction, Slc25a4^K147E^ -expressing cells exhibited significantly fewer Alp^+^ osteoblasts and downregulated expression of key osteogenic markers compared to Slc25a4^K147R^ (Fig. 5h, i and Fig. S7b). By day 21, Slc25a4^K147E^ expression markedly reduced mineralization capacity (Fig. 5h and Fig. S7b). These findings suggest that Slc25a4 K147 succinylation potently suppresses BMSCs osteogenic differentiation. To elucidate the mechanistic basis of osteogenic inhibition, we compared mitochondrial function in Slc25a4^K147R^- versus Slc25a4^K147E^-expressing BMSCs after 7-day differentiation. Seahorse analysis revealed Slc25a4^K147E^ significantly impaired respiratory capacity, reducing ATP production, basal OCR, and maximal respiration (Fig. 5j and Fig. S7c), while elevating intracellular ROS levels (Fig. 7d). Flow cytometric analysis confirmed a significantly higher apoptosis rate in Slc25a4^K147E^-expressing cultures (Fig. S7e). These findings demonstrate that Slc25a4 K147 succinylation drives osteoporotic bone loss by suppressing osteogenic differentiation and promoting apoptosis through mitochondrial dysfunction and ROS accumulation.

### Sirt5-dependent osteogenesis requires Slc25a4 desuccinylation

**Fig. 6:**
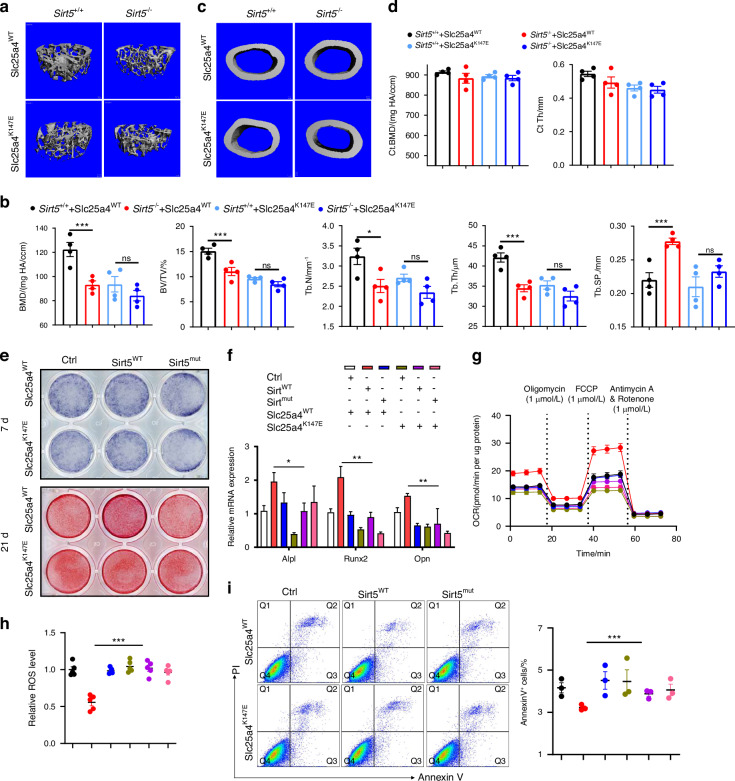
Sirt5 promotes osteogenesis depending on desuccinylating Slc25a4. **a** 3D micro-CT reconstruction images of trabecular bone in *Sirt5*^+/+^ or *Sirt5*^-/-^ mice overexpressed Slc25a4^WT^ or Slc25a4^K147E^. **b** Quantitative microarchitectural parameters of micro-CT. (n = 4) **c** 3D micro-CT reconstruction images of cortical bone. **d** Quantitative microarchitectural parameters of micro-CT. **e** Upper: Alp staining in BMSCs overexpressing Ctrl+Slc25a4^WT^, Sirt5^WT^+Slc25a4^WT^, Sirt5^mut^+Slc25a4^WT^, Ctrl+Slc25a4^K147E^, Sirt5^WT^+Slc25a4^K147E^, and Sirt5^mut^+Slc25a4^K147E^, after 7-day osteogenic differentiation. Lower: Alizarin red staining after 21-day osteogenic differentiation. Relative mRNA levels of osteogenic markers (**f**), mitochondrial respiration (OCR; **g**), intracellular ROS levels (**h**), and apoptosis analysis by flow cytometry (**i**). Data represent mean ± SEM in (**b**, **d**, **f**–**i**). Statistics used an unpaired two-tailed *t*-test (**b**, **d**, **f**–**i**). Significance is noted as *P < 0.05, **P < 0.01, ***P < 0.001

Our findings demonstrate that Slc25a4 K147 succinylation drives osteoporotic bone loss through mitochondrial dysfunction-induced ROS accumulation and osteocyte apoptosis. To determine whether Sirt5-mediated protection against osteoporosis specifically requires Slc25a4 desuccinylation, we performed genetic complementation experiments in Sirt5-deficient and wild-type backgrounds. Methodologically, we overexpressed Slc25a4^WT^ and Slc25a4^K147E^ in bone marrow in *Sirt5*^-/-^ and *Sirt5*^+/+^ mice, followed by OVX to induce osteoporosis. After 30 days, we collected femora for micro-CT analysis. The results indicated that when Slc25a4^WT^ was overexpressed, the trabecular mass and bone formation rate in *Sirt5*^-/-^ mice were significantly lower than those in *Sirt5*^+/+^ mice. Conversely, when Slc25a4^K147E^ was overexpressed and Slc25a4 could not be de-succinylated, the trabecular mass and bone formation rate in *Sirt5*^-/-^ and *Sirt5*^+/+^ mice were reduced, with no statistically significant differences observed between the two groups (Fig. 6a, b). Additionally, there were no significant changes in cortical mass (Fig. 6c, d). These findings conclusively demonstrate that Sirt5 exerts its anti-osteoporotic effects primarily through enzymatic desuccinylation of Slc25a4 at K147.

We identify Slc25a4 succinylation as a key mechanism through which Sirt5 modulates BMSCs differentiation and apoptosis. To determine whether this regulation depends specifically on Sirt5-mediated desuccinylation of Slc25a4, we conducted combinatorial genetic experiments in BMSCs. We established six experimental groups through co-overexpression of different Sirt5 and Slc25a4 variants: Ctrl+Slc25a4^WT^, Sirt5^WT^+Slc25a4^WT^, Sirt5^mut^+Slc25a4^WT^, Ctrl+Slc25a4^K147E^, Sirt5^WT^+Slc25a4^K147E^, and Sirt5^mut^+Slc25a4^K147E^. Following osteogenic induction, we observed: at day 7, the Sirt5^WT^+Slc25a4^WT^ group showed significantly more Alp-positive osteoblasts and higher Alp activity than Sirt5^WT^+Slc25a4^K147E^ (Fig. 6e and Fig. S7f), and at day 21, enhanced mineralization in Sirt5^WT^+Slc25a4^WT^ versus Sirt5^WT^+Slc25a4^K147E^ (Fig. 6e and Fig. S7f). RNA was extracted and analyzed after 7 days of differentiation. The results indicated that the Sirt5^WT^+ Slc25a4^WT^ group exhibited an increased expression of osteogenesis-related genes (Fig. 6f). These results establish that Sirt5 promotes osteogenesis specifically through its enzymatic activity in desuccinylating Slc25a4.

To investigate the role of Slc25a4 succinylation in Sirt5’s regulation of mitochondrial function and apoptosis, we overexpressed various mutants of Sirt5 and Slc25a4, categorizing them into 6 distinct groups in BMSCs, and performed osteogenic differentiation for 7 days. Seahorse assays indicated an increase in mitochondrial OCR (Fig. 6g and Fig. S7g) and a decrease in intracellular ROS levels in Sirt5^WT^+Slc25a4^WT^ group compared to Sirt5^WT^+Slc25a4^K147E^ group (Fig. 6h). Furthermore, flow cytometry analysis was employed to assess the percentage of apoptotic cells, revealing that Sirt5^WT^+Slc25a4^WT^ group significantly decreased the proportion of apoptotic cells (Fig. 6i). These data indicate that the effects of Sirt5, which promote osteogenic differentiation and inhibit apoptosis by enhancing mitochondrial function and reducing ROS levels, depend on its desuccinylation of the Slc25a4 site at K147.

## Discussion

Osteoporosis pathogenesis involves a critical imbalance between osteogenic and osteoclastogenic activities,^33^ with the regulatory mechanisms of osteogenesis remaining relatively understudied. Our integrated multi-omics approach, combining analysis of osteogenic differentiation transcriptomes and osteoporosis patient expression profiles with ROC validation, identified two key findings: significant alterations in pyruvate metabolism during osteogenic differentiation and Sirt5 as a robust predictor of osteoporosis risk. Given Sirt5’s established role in mitochondrial regulation and these preliminary findings, we focused our investigation on elucidating Sirt5’s biological functions and molecular mechanisms in regulating osteogenesis through mitochondrial pathways.

To elucidate the role of Sirt5 in osteoporosis pathogenesis, we employed an ovariectomized Sirt5 knockout mouse model to accelerate bone loss and establish osteoporosis. Our findings demonstrate that Sirt5 exerts protective effects against osteoporosis development. In vitro studies revealed that this protection is mediated through enhanced osteogenic differentiation. Given that Sirt5 functions through its catalytic domain to mediate desuccinylation and deacetylation, we generated an enzymatically inactive mutant (H158Y)^27^ to verify the requirement of its catalytic activity. The inability of this mutant to promote osteogenesis confirmed that Sirt5’s regulatory effects are strictly dependent on its enzymatic function.

Integrating our observations of altered pyruvate metabolism during osteogenic differentiation with Sirt5’s known mitochondrial regulatory role, we examined mitochondrial respiration in BMSCs. Sirt5 deficiency resulted in impaired respiratory capacity and elevated ROS levels, creating a cellular environment that both suppresses osteogenic differentiation and promotes apoptosis. This dual mechanism ultimately contributes to the development of osteoporotic bone loss.

Through systematic analysis of protein post-translational modifications during osteogenesis, we identified Sirt5 as a primary regulator of global succinylation levels and discovered Slc25a4 as its key functional target. As an essential mitochondrial inner membrane protein,^34^ Slc25a4 maintains membrane potential, and its abnormal accumulation increases membrane permeability, impairing respiration and elevating ROS.^35^ Our interaction studies and functional validation demonstrate that Sirt5 promotes Slc25a4 degradation through K147 desuccinylation, thereby preserving mitochondrial function. This regulatory mechanism maintains proper respiratory capacity and redox balance while facilitating osteogenic differentiation, establishing that Sirt5 governs osteogenesis through mitochondrial regulation via site-specific desuccinylation of Slc25a4.

In summary, our study reveals that Sirt5-mediated desuccinylation of Slc25a4 at K147 constitutes a critical regulatory mechanism in osteoporosis pathogenesis. We demonstrate that this specific post-translational modification serves as a molecular switch governing mitochondrial oxidative phosphorylation and osteogenic differentiation in bone marrow stromal cells. Specifically, Sirt5-dependent removal of succinylation at K147 promotes Slc25a4 degradation, thereby maintaining mitochondrial function and preventing ROS accumulation. These findings not only establish the Sirt5-Slc25a4-K147 axis as a central regulator of bone homeostasis but also identify this specific lysine modification as a potential therapeutic target for modulating bone formation in osteoporosis.

## Methods and materials

### *Sirt5*^-/-^ mice model

All animal research was conducted using the “Guidelines for the Care and Use of Laboratory Animals” (Ministry of Science and Technology of China, 2006) and the relevant ethical regulations of the hospital. All experimental procedures received approval from the hospital’s Animal Care and Use Committee. The animals were housed at room temperature (22 °C–25 °C), with unrestricted access to water and food, and were subjected to a daily 12-h light/dark cycle.

*Sirt5* knockout mice were generated using the CRISPR-Cas9 system. *Sirt5*-specific single-guide RNA (sgRNA) was designed, synthesized, and mixed with Cas9 mRNA at a concentration of 100 ng/μL. This mixture, containing sgRNA at 50 ng/μL, was injected into the fertilized eggs of mice via microinjection. The injected fertilized eggs were cultured to the two-cell stage at 37 °C in a 5% CO_2_ atmosphere, and then transplanted into the fallopian tubes of pseudopregnant female mice. After the newborn pups were weaned, tail DNA was extracted, and the efficacy of gene editing was verified using polymerase chain reaction (PCR) and Sanger sequencing.

### OVX-mice model

Six-week-old female mice underwent ovariectomy to establish an ovariectomy-induced osteoporosis model. After the procedure, the mice were randomly divided into four experimental groups. After 40 days, the mice were euthanized and samples were collected for subsequent analysis.

### Micro-CT analysis

The femora from experimental mice were dissected and preserved in ethanol before being scanned using a micro-CT scanner. A 1-mm-wide section of trabecular bone adjacent to the distal growth plate of the femora was three-dimensionally reconstructed and analyzed for microarchitectural parameters, including BMD, BV/TV, Tb.Th., Tb.N., and Tb.Sp. Additionally, a 1-mm-wide section of cortical bone from the midshaft of the femora was evaluated for Ct.BMD and Ct.Th.

### Hematoxylin and eosin (H&E) staining

The distal femoral tissues were fixed in 4% paraformaldehyde for 48 h and subsequently treated with 15% ethylenediaminetetraacetic acid. Paraffin-embedded sections were then stained with hematoxylin and eosin according to the following procedure.

### Immunohistochemistry (IHC)

Tissue sections were sequentially rehydrated using xylene and anhydrous ethanol solutions of varying concentrations. Subsequently, the sections underwent antigen retrieval by boiling in a sodium citrate antigen recovery solution at temperatures ranging from 95 °C to 100 °C for 10 min. Endogenous peroxidase activity was then inactivated using a 3% hydrogen peroxide solution. The sections were incubated overnight at 4 °C with an anti-Alp antibody, followed by incubation with a secondary antibody. The DAB staining kit was employed according to the manufacturer’s instructions. After counterstaining with hematoxylin, the sections were dehydrated sequentially with anhydrous ethanol and xylene solutions of varying concentrations, and subsequently mounted with a sealing medium. Microscopic analysis was performed using a Zeiss AXIO BX61 microscope.

### Spinal cord injection of adeno-associated virus

Following the administration of anesthesia, the dorsal fur of the mice was shaved, and a ¼ gauge needle was inserted into the lumbar spine at a 70-degree angle along the midline. Upon contact with the bone, the angle of the needle was adjusted to 30°, allowing for insertion between the vertebral segments. The plunger of the syringe was then gently depressed to inject 5–10 μL of adeno-associated virus (AAV) suspension into the spinal cord. After the injection, the needle was rotated 180 degrees one to two times before being withdrawn from the spine. The mice were subsequently returned to their cages for observation, during which normal motor function was confirmed.

### Three-point bending test

Femora were collected and preserved in 75% ethanol. Three-point bending tests were conducted using an Instron universal testing machine (Instron, Canton, MA) at the midshaft of the femora, with a displacement rate of 0.03 mm/s until fracture occurred. The maximum load was determined from load-deflection diagrams.

### Calcein labeling

Mice received intraperitoneal injections of 20 mg/kg calcein (1 mg/mL in a 2% NaHCO_3_ solution) on days 0 and 4. The mice were sacrificed on day 7, and their tibiae were isolated, dehydrated, and embedded in polymethylmethacrylate. Samples were sectioned into 5-μm-thick slices using a hard tissue cutter (RM2265, Leica, Wetzlar, Germany). Fluorescence-labeled images were captured using a microscope (BX51, Olympus).

### Cell culture, transfection, and viral infection

The femora of the hind limbs from 4-week-old male mice were isolated, and the bone marrow was extracted on the 4th day of culture. Minimum Essential Medium Alpha (MEMα) was subsequently added to provide nutritional supplementation. The cells were utilized after the second passage generation had commenced.

Transfection was performed using Lipofectamine™ 3000 Transfection Reagent (Invitrogen, L3000015) according to the manufacturer’s instructions. To generate reconstituted cells, BMSCs were transfected with the appropriate plasmid. After a 48-h transfection period, the supernatant was replaced with either growth medium or differentiation medium.

### Alp staining

Utilize the BCIP/NBT Alkaline Phosphatase Color Development Kit from Beyotime (C3206) for the staining procedure. Sequentially add each solution in the specified proportions and mix thoroughly to prepare an adequate volume of the BCIP/NBT staining working solution. Subsequently, remove the cell culture medium from the samples to be stained and add 4% paraformaldehyde for fixation, allowing it to incubate for 30 min. After discarding the fixation solution, wash each well twice with PBS. Next, add 500 μL of the BCIP/NBT staining solution to ensure complete coverage of the samples and incubate at room temperature for 30 min in the absence of light. Following incubation, remove the BCIP/NBT staining solution, add 500 μL of washing solution to each well, and perform two additional washes. Finally, observe the samples under a microscope and proceed to scan and photograph the results.

### Alp activity

We utilized the Beyotime Alkaline Phosphatase Assay Kit (P0321S) for this experiment. First, dilute the test samples and prepare a series of standard concentrations. Next, add 100 μL of the test samples or standards to each well of a 96-well plate, followed by the addition of 50 μL of alkaline phosphatase substrate solution (p-NPP). Gently mix the contents and incubate the plate at 37 °C for 30 min, ensuring that the plate is protected from light exposure. After incubation, add 50 μL of 1 mol/L NaOH solution to each well to terminate the reaction, and mix gently once more. Finally, measure the absorbance values at 405 nm using a microplate reader and record the absorbance for each well. Plot a standard curve based on the absorbance values of the standards, and use this curve to calculate the alkaline phosphatase activity in the test samples.

### Alizarin red staining

After 21 days of osteogenic differentiation, the culture medium was aspirated, and the cells were fixed with 4% paraformaldehyde for 15 min, followed by two washes with PBS. Subsequently, 1 mL of 40 mmol/L alizarin red dye was added to each well, and the samples were incubated at room temperature for 10 min with gentle agitation. The unbound dye was then removed, and 1 mL of ddH_2_O was added to cover the cells. The cells were washed four times on a shaker, with each wash lasting 5 min. The results were documented through scanning and photography.

### Liquid chromatography-tandem mass spectrometry (LC-MS/MS) analysis

LC-MS/MS analysis of peptide succinylation products was performed and analyzed by APTBIO (Shanghai, China). Proteins were extracted from 7-day differentiated BMSCs using a buffer containing 8 mol/L urea and 100 mmol/L Tris/HCl (pH 8.5), followed by quantification using the Bradford method. After SDS-PAGE and Coomassie blue staining, proteins (20 µg) were reduced with dithiothreitol (DTT), alkylated with iodoacetamide (IAA), and digested overnight with trypsin at 37 °C. The resulting peptides were desalted using C18 cartridges and lyophilized. Succinylated peptides were enriched using anti-succinyl-lysine antibody beads and further desalted with C18 STAGE Tips. Peptide samples were separated on an Easy nLC system with a gradient of 0.1% formic acid in water and 0.1% formic acid in acetonitrile, utilizing Thermo Scientific Acclaim PepMap 100 and EASY columns. The separated peptides were analyzed on a timsTOF Pro mass spectrometer in positive ion mode, with an ion source voltage of 1.5 kV, scanning from 100 to 1 700 m/z. Data acquisition was performed using PASEF mode. Raw mass spectrometry data were processed for identification and quantification using Peaks software.

### Flow cytometry

The cells were incubated with propidium iodide (PI) (BD Biosciences) and FITC Annexin V (BD Biosciences) for 30 min in the dark at room temperature. The percentage of apoptosis in BMSCs was assessed using a flow cytometer. The resulting data were analyzed using FlowJo software version 1.8.0.

### Immunoblotting analysis

The cells were lysed using a lysis solution, and the proteins were subsequently separated by SDS-PAGE and transferred to PVDF membranes. To minimize nonspecific background, a buffer containing 5% milk powder was utilized. Protein bands were detected using various antibodies, as specified. The membranes were incubated with primary antibodies at 4 °C overnight, followed by incubation with secondary antibodies for 1 h at room temperature before detection with an AI600 system in a dark environment. The catalog numbers of the primary antibodies are provided in Supplementary Table 1.

### Coimmunoprecipitation

HEK293T cells were co-transfected with plasmids encoding Sirt5 and Slc25a4 and cultured for 48 h. The cells were lysed using IP lysis buffer and incubated overnight at 4 °C with anti-Sirt5 and anti-Slc25a4 antibodies. Protein A/G magnetic beads were then added to the immune complex solution and incubated at room temperature for 1 h, followed by washing to remove any unbound immune complexes. The bound immune complexes were separated from the magnetic beads using an ice-cold buffer for Western blot analysis. The antibodies utilized in this study are listed in Supplementary Table 1.

### RNA sequencing experiment and analysis

Total RNA was extracted from *Sirt5*^+/+^ and *Sirt5*^-/-^ BMSCs, and a complementary DNA (cDNA) library was constructed following the standard protocols outlined by Illumina for RNA-seq. The software FeatureCounts version 1.5.0-p3 was employed to quantify the number of reads mapped to each gene. A fold change > 1.5 and *P* < 0.05 were set as the thresholds for identifying the DEGs. GO enrichment analysis and KEGG analysis of differentially expressed genes were performed using the “clusterProfiler” R package.

### Reverse-transcription polymerase chain reaction and quantitative PCR assays

Quantitative PCR was conducted utilizing an ABI Prism 7300 system (Applied Biosystems, Foster City, CA, USA) in conjunction with SYBR Green (Takara, Dalian, China). For the PCR process, a maximum of 1 μL of cDNA was employed as the template. The thermal cycling parameters consisted of an initial denaturation at 95 °C for 10 s, followed by 40 cycles of denaturation at 95 °C for 5 s and annealing/extension at 60 °C for 30 s. The efficiency of the primers was determined to be greater than 90%, as confirmed by a standard curve that spanned four orders of magnitude. After the reactions, the raw data were exported using the 7300 System Software version 4 v1.3.0 (Applied Biosystems) for analysis. The primers utilized in this study are detailed in Supplementary Table 2.

### Oxygen consumption rate (OCR) by Seahorse assay

Cellular OCR was measured by the XFe96 Extracellular Flux Seahorse Analyzer (Agilent, USA). In brief, cells were inoculated into XFe96 cell culture microplates and allowed to incubate overnight. During the assay, various compounds were introduced into designated ports: Port A received Oligomycin at a final concentration of 1 μmol/L; Port B was treated with FCCP at a final concentration of 1 μmol/L; and Port C was supplied with Antimycin A and Rotenone, also at a final concentration of 1 μmol/L. The experiment was conducted on the Agilent Seahorse XFe96, utilizing Wave Desktop and Report Generator software for data visualization and analysis.

### Detection of ROS

After treating the cells according to the experimental design, aspirate the culture medium and wash the cells twice with PBS for 5 min. Add 100 μL of DCFH-DA solution to each well and incubate at 37 °C in the dark for 30 min. Remove the staining solution and wash the cells three times with PBS for 5 min to remove unbound probe. Add 100 μL of fresh PBS to each well and measure the fluorescence intensity using a microplate reader at 488 nm excitation and 525 nm emission. Record the fluorescence values (Thermo Fisher Scientific, C10444).

### WGCNA network construction and module identification

The GSE80614 and GSE100752 datasets were sourced from the Gene Expression Omnibus (GEO: https://www.ncbi.nlm.nih.gov/geo/). Data cleaning, organization, error checking, and statistical analysis were conducted using the “Limma” R package. Following this, the “WGCNA” R package was employed to construct the co-expression network, which facilitated the identification of the associated gene modules.

### Receiver operating characteristic (ROC) curve and plot

The GSE30159 dataset was obtained from the GEO. Data cleaning, organization, error checking, and statistical analysis were performed utilizing the “Limma” R package. A binary value was assigned based on the presence or absence of osteoporosis in the patients. Subsequently, the “ROCR” R package was employed to develop the ROC prediction model, and the ROC curve was generated.

### Statistical analysis

The investigators-maintained blinding to group allocation throughout the experimental procedures of the study. Statistical analyses were performed using GraphPad Prism 8. Data are presented as the mean ± standard error of the mean (SEM). The correlation was evaluated using Pearson correlation analysis. A one-way analysis of variance (ANOVA) was employed to assess the significance of differences among groups. Student’s *t*-test was utilized for pairwise comparisons between groups. A two-sided *P* - value of less than 0.05 was considered statistically significant. Sample sizes were determined based on prior publications, without conducting a preliminary power analysis.

## Supplementary information

Supplementary Information

## Data Availability

The RNA-seq data generated in this study are publicly available in the Genome Sequence Archive under accession numbers CRA027018 (https://ngdc.cncb.ac.cn/gsa/s/HFn15PT0). Publicly available datasets analyzed in this study can be found in the Gene Expression Omnibus under accession numbers GSE80614, GSE100752, and GSE30159. Nonprofit research will be approved for access to the newly generated data. All other raw data are available upon request from the corresponding author.
