# Supplementary material for: Sirtuin 5-mediated desuccinylation of Slc25a4 inhibits osteoporosis by enhancing mitochondrial respiration
Source: Bone Res. 2025 Nov 17;13:93. doi: 10.1038/s41413-025-00464-7 (PMC12620516; doi:10.1038/s41413-025-00464-7)
Supplement: Supplementary file 1 — Supplementary Information [file 41413_2025_464_MOESM1_ESM.docx]

**Supplementary information**

**Sirtuin 5-mediated desuccinylation of Slc25a4 inhibits osteoporosis by enhancing mitochondrial respiration**

Jun Chen^1,2,3,4^, Xinquan Jiang^1,2,3,4 *^

1. Department of Prosthodontics, Shanghai Ninth People's Hospital, Shanghai Jiao Tong University School of Medicine, No. 639 Zhizaoju Road, Shanghai, 200011, China.

2. Shanghai Stomatological Hospital, Fudan University, No.166 Hechuan Road, Shanghai, 200000, China.

3. College of Stomatology, Shanghai Jiao Tong University, No. 115 Jinzun Road, Shanghai, 200125, China.

4. National Center for Stomatology, National Clinical Research Center for Oral Diseases, Shanghai Key Laboratory of Stomatology, Shanghai Research Institute of Stomatology, Shanghai Engineering Research Center of Advanced Dental Technology and Materials, No. 639 Zhizaoju Road, Shanghai, 200011, China.

*Corresponding authors: xinquanjiang@aliyun.com

**This file includes:**

Supplementary Fig. 1. WGCNA of osteogenic differentiation transcriptomes

Supplementary Fig. 2. Transcriptomic profiling of osteogenic differentiation regulators

Supplementary Fig. 3. Diagnostic performance of the osteogenic dysfunction prediction model

Supplementary Fig. 4. Functional characterization of Sirt5 in osteogenic differentiation and mitochondrial metabolism

Supplementary Fig. 5. Succinylome profiling during osteogenic differentiation

Supplementary Fig. 6. Proteomic and functional analysis of SIRT5-regulated pathways

Supplementary Fig. 7. Functional validation of Slc25a4 K147 modification in osteogenesis and mitochondrial metabolism

Supplementary Table 1. Antibodies used in the project

Supplementary Table 2. The primers used for quantitative RT-PCR


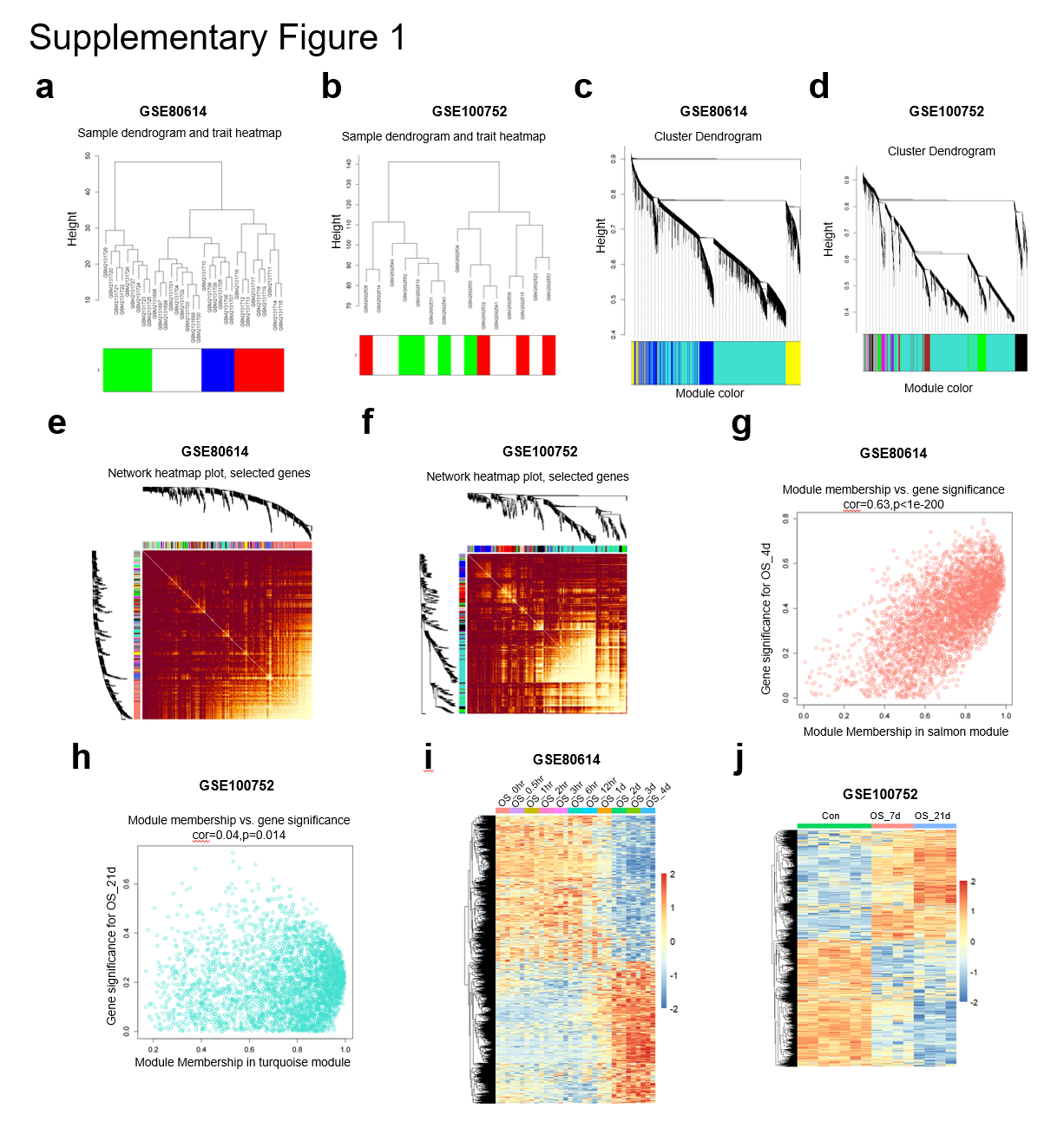


**Supplementary Fig. 1** (a) and (b) Construction of the co-expression network in the GSE80614 and GSE100752 datasets. All samples are located in the clusters and pass the cutoff thresholds. (c) and (d) Hierarchical cluster analysis was conducted to detect co-expression clusters with corresponding color assignments. Each color represents a module in the constructed gene co-expression network by WGCNA. (e) and (f) Heatmap depicts the TOM of genes selected for weighted co-expression network analysis. Light color represents lower overlap, and red represents higher overlap. (g) and (h) Scatter plot describing the relationship between MM and GS for the salmon module in the GSE80614 and GSE100752 datasets. (i) and (j) Heatmap of the salmon module in the GSE80614 and GSE100752 datasets.


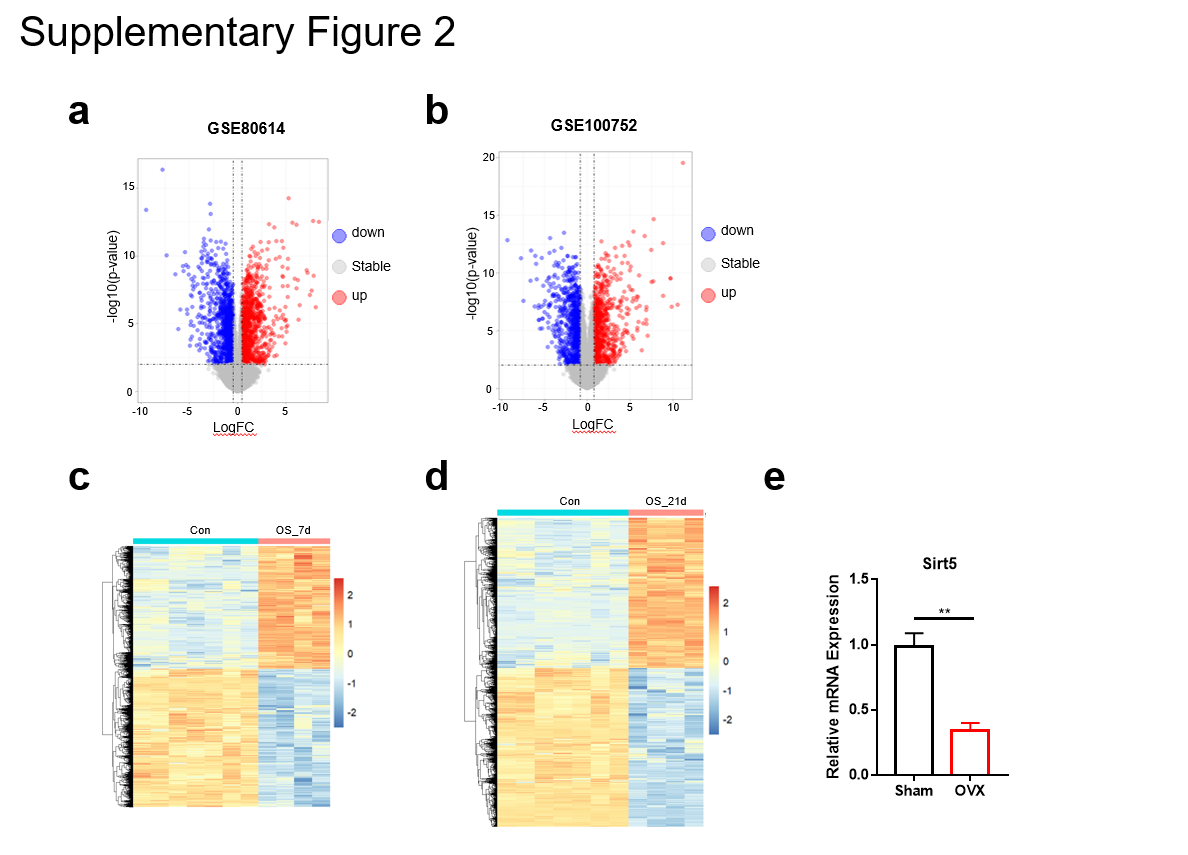


**Supplementary Fig. 2** (a) and (b) Volcano plot of DEG showing genes from the GSE100752 dataset. (c) and (d) Heatmap of DEG showing genes from GSE100752 datasets. (e) Relative expression levels of *Sirt5* in BMSCs of Sham or OVX mice. Data represent mean ± SEM. Statistics used a unpaired two-tailed t-test. Significance is noted as ∗∗p < 0.01.


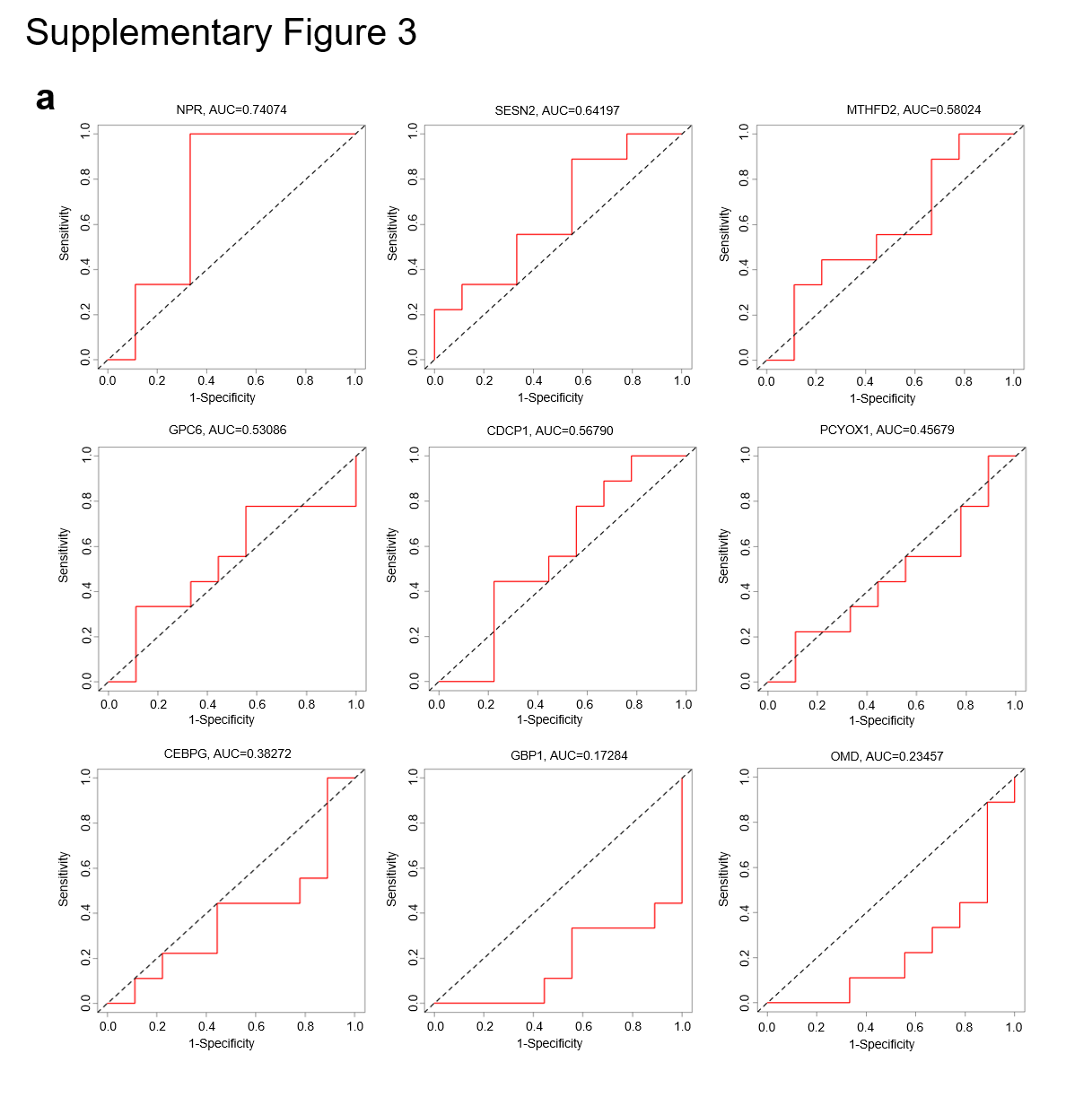


**Supplementary Fig. 3** a. ROC curve of the disease prediction model based on nine biomarkers (NPR3, SESN2, MTHFD2, GPC6, CDCP1, PCYOX1, CEBPG, GBP1, and OMD) using the GSE30159 dataset.


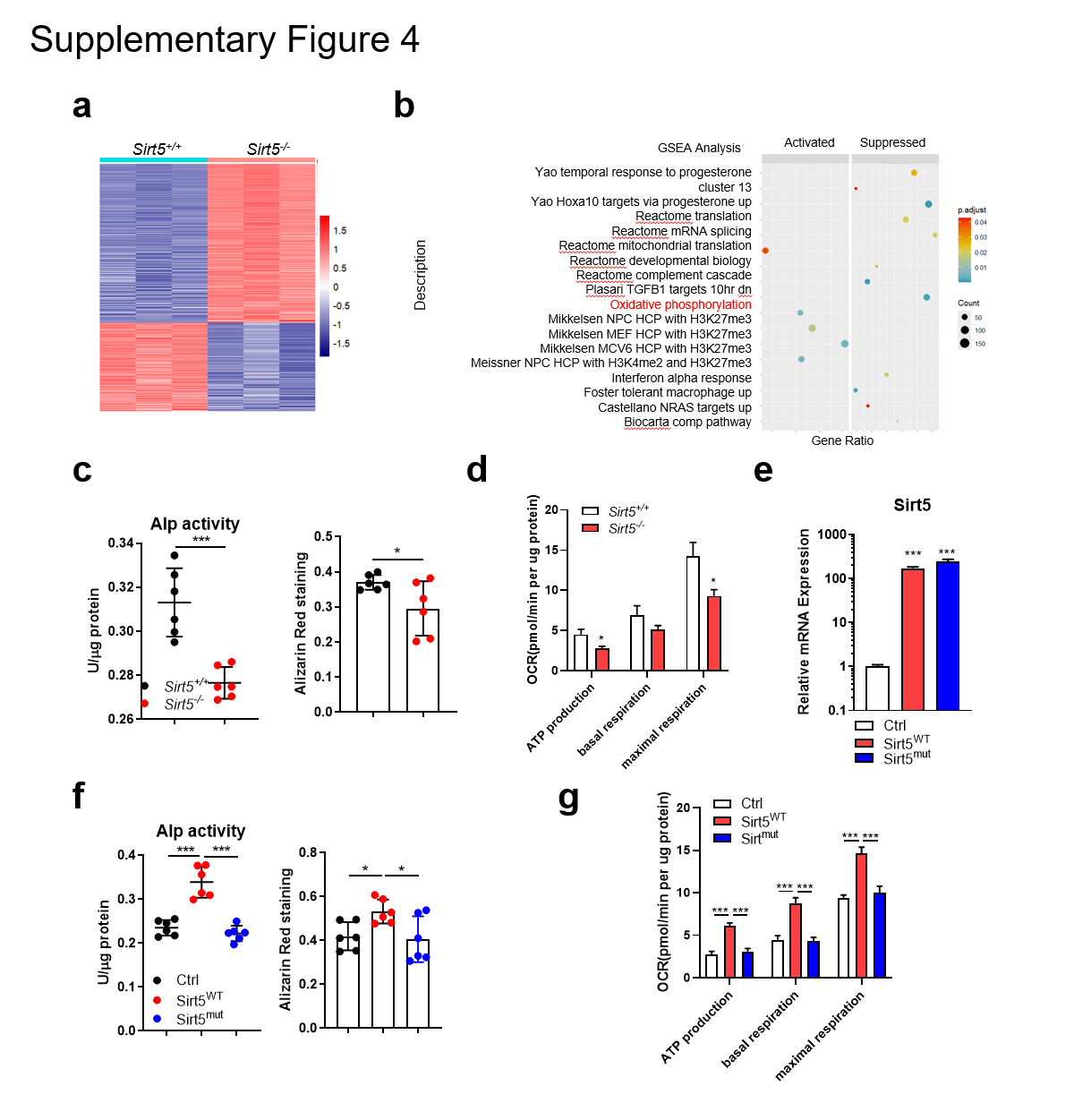


**Supplementary Fig. 4** (a) Heatmap of DEGs in *Sirt5*^+/+^ and *Sirt5*^-/-^ BMSCs after 7-day osteogenic differentiation. (b) GSEA showing the most significant altered pathways. (c) Left: Alp activity in *Sirt5*^+/+^ and *Sirt5*^-/-^ BMSCs after 7-day osteogenic differentiation. Right: quantitative analysis of Alizarin red staining after 21-day osteogenic differentiation. (d) Quantification of OCR in *Sirt5*^+/+^ and *Sirt5*^-/-^ BMSCs. (e) Relative expression levels of BMSCs overexpressing Ctrl, Sirt5^WT^, or Sirt5^mut^ after 7-day osteogenic differentiation. (f) Left: Alp activity in BMSCs overexpressing Ctrl, Sirt5^WT^, or Sirt5^mut^. Right: quantitative analysis of Alizarin red staining of BMSCs. (g) Quantification of OCR in BMSCs. Data represent mean ± SEM in c-g. Statistics used an unpaired two-tailed t-test (c-g). Significance is noted as ∗p < 0.05, ∗∗∗p < 0.001.


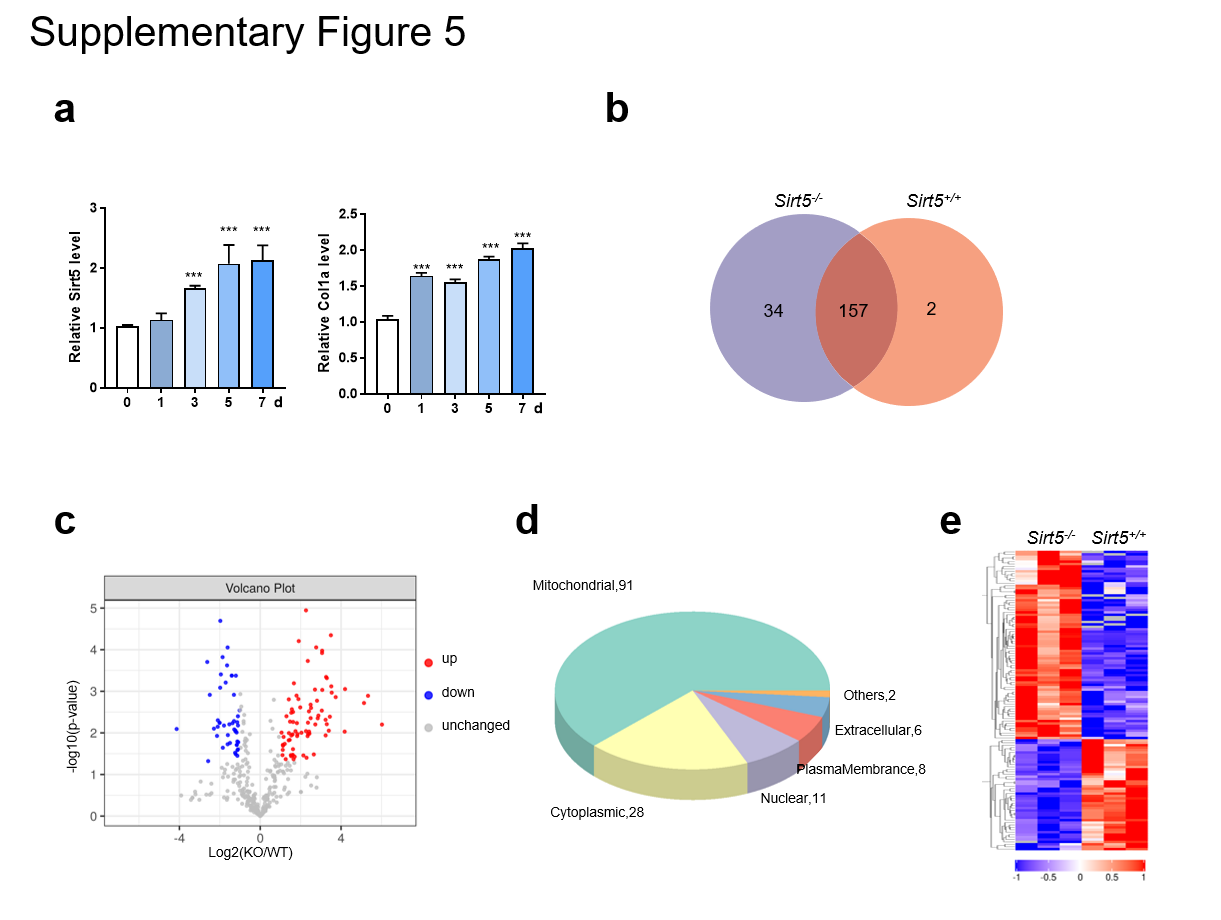


**Supplementary Fig. 5** (a) Quantification of SIRT5 and COL1A1 expression in BMSCs during osteogenic induction. (n = 3) (b) Venn diagram showing the numbers of succinylated proteins in *Sirt5*^+/+^ and *Sirt5*^-/-^ BMSCs. (c) Volcano plot of differential protein expression in *Sirt5*^-/-^ compared to *Sirt5*^+/+^ BMSCs. (d) Pie chart showing location of proteins in *Sirt5*^-/-^ compared to *Sirt5*^+/+^ BMSCs. (e) Heatmap of succinylated proteins in *Sirt5*^+/+^ and *Sirt5*^-/-^ BMSCs. Data represent mean ± SEM in a. Statistics used an unpaired two-tailed t-test (a). Significance is noted as ∗∗∗p < 0.001.


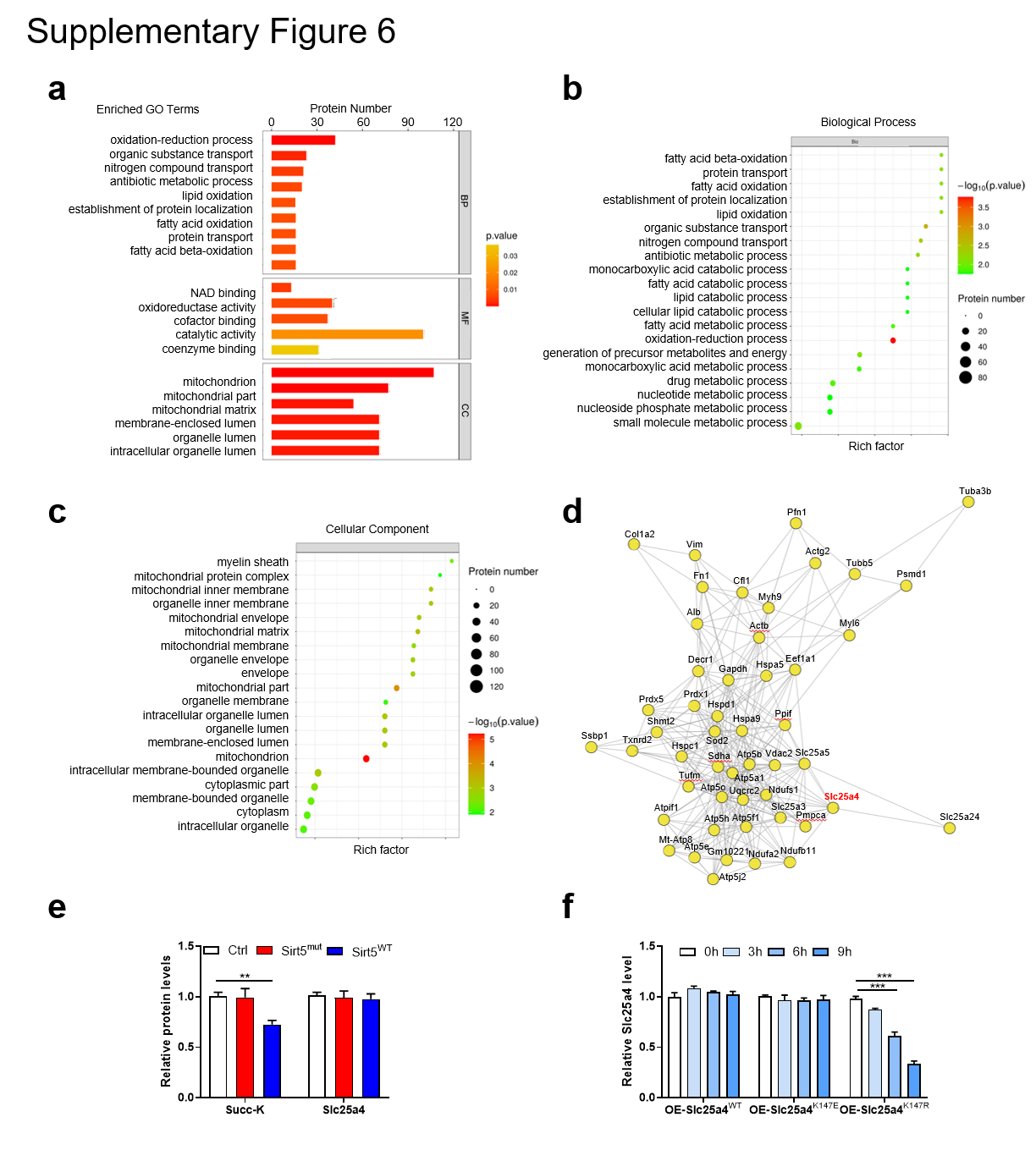


**Supplementary Fig. 6** (a) GO analysis of differentially expressed proteins in *Sirt5*^-/-^ compared to *Sirt5*^+/+^ BMSCs. (b) and (c) GO enrichment of biological processes and cellular components among dysregulated proteins. (d) Protein-protein interaction network of SIRT5-associated targets made in STRING. (e) Quantification of Succ-K and SLC25A4 levels in whole-cell lysates and immunoprecipitates from HEK293 cells expressing Ctrl, Sirt5^WT^, or Sirt5^mut^ for 48 hours. (n = 3) (f) Quantification of Slc25a4 expression in whole cell lysates from HEK293 cells expressing Slc25a4^WT^, Slc25a4^K147E^, or Slc25a4^K147R^ after treatment with CHX for 0, 3, 6, and 9 hours. (n = 3) Data represent mean ± SEM in e and f. Statistics used unpaired two-tailed t-test (e and f). Significance is noted as ∗∗p < 0.01, ∗∗∗p < 0.001.


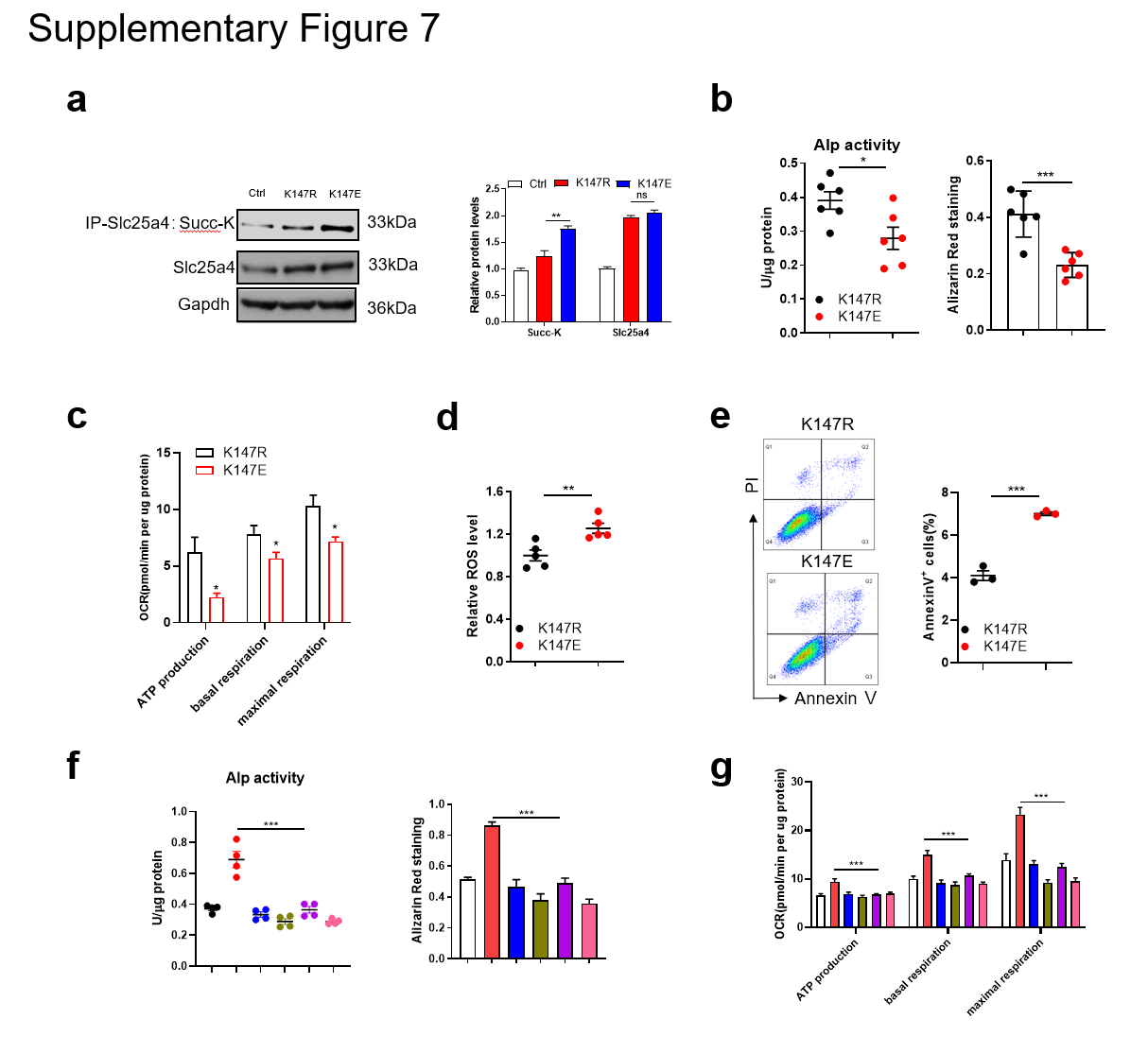


**Supplementary Fig. 7** (a) Immunoblotting against Gapdh and Slc25a4 in femoral lysates 7 days post-injection and Succ-K levels in SLC25A4 immunoprecipitates. (b) Left: Alp activity in BMSCs overexpressing Slc25a4^K147R^ or Slc25a4^K147E^ after 7-day osteogenic differentiation. Right: quantitative analysis of Alizarin red staining after 21-day osteogenic differentiation. (c-e) Quantitative of OCR (c), ROS levels (d), and apoptosis analysis by flow cytometry (e) in BMSCs overexpressing Slc25a4^K147R^ or Slc25a4^K147E^ after 7-day osteogenic differentiation. (f) Left: Alp activity in BMSCs overexpressing Ctrl+Slc25a4^WT^, Sirt5^WT^+Slc25a4^WT^, Sirt5^mut^+Slc25a4^WT^, Ctrl+Slc25a4^K147E^, Sirt5^WT^+Slc25a4^K147E^, and Sirt5^mut^+Slc25a4^K147E^, after 7-day osteogenic differentiation. Right: quantitative analysis of Alizarin red staining of BMSCs after 21-day osteogenic differentiation. (g) Quantification of OCR in BMSCs. Data represent mean ± SEM in a-g. Statistics used unpaired two-tailed t-test (a-g). Significance is noted as ∗p < 0.05, ∗∗p < 0.01, ∗∗∗p < 0.001.

**Supplementary Table1**

| Ab for immunoblotting | Source | Catalog Number |
| --- | --- | --- |
| Sirt5 | ABclonal | A23083 |
| Succinylatied-Lysine | ThermoFisher | PA5-120740 |
| Acetylated-Lysine | Cell Signaling Technology | 9441 |
| Alpl | ABclonal | A0514 |
| Col1a  Gapdh | ABclonal  ABclonal | A26946  AC002 |
| Slc25a4 | ABclonal | A15027 |

**Supplementary Table2**

| Gene | Forward primer (5'-3') | Reverse primer (5'-3') |
| --- | --- | --- |
| Gapdh | AATGGATTTGGACGCATTGGT | TTTGCACTGGTACGTGTTGAT |
| Sirt5 | CCAGTTGTGTTGTAGACGAAAGC | TTCCGAAAGTCTGCCATATTTGA |
| Opn | ATCTCACCATTCGGATGAGTCT | TGTAGGGACGATTGGAGTGAAA |
| Runx2 | GACTGTGGTTACCGTCATGGC | ACTTGGTTTTTCATAACAGCGGA |
| Alpl | GGCTGGAGATGGACAAATTCC | CCGAGTGGTAGTCACAATGCC |
| Bglap | CTGACCTCACAGATCCCAAGC | TGGTCTGATAGCTCGTCACAAG |
